# Effects of Sugarcane and Soybean Intercropping on the Nitrogen-Fixing Bacterial Community in the Rhizosphere

**DOI:** 10.3389/fmicb.2021.713349

**Published:** 2021-09-30

**Authors:** Yue Liu, Wenqing Ma, Hongliang He, Ziting Wang, Yanhong Cao

**Affiliations:** ^1^College of Agronomy, Guangxi University, Nanning, China; ^2^Guangxi South Subtropical Agricultural Science Research Institute, Chongzuo, China; ^3^Guangxi Key Laboratory of Livestock Genetic Improvement, The Animal Husbandry Research Institute of Guangxi Zhuang Autonomous Region, Nanning, China

**Keywords:** sugarcane, nitrogen-fixing bacteria, intercropping, network analysis, rhizosphere

## Abstract

Intercropping between sugarcane and soybean is widely used to increase crop yield and promote the sustainable development of the sugarcane industry. However, our understanding of the soil microenvironment in intercropping systems, especially the effect of crop varieties on rhizosphere soil bacterial communities, remains poor. We selected two excellent sugarcane cultivars, Zhongzhe1 (ZZ1) and Zhongzhe9 (ZZ9), from Guangxi and the local soybean variety GUIZAO2 from Guangxi for field interplanting experiments. These two cultivars of sugarcane have good drought resistance. Rhizosphere soil samples were collected from the two intercropping systems to measure physicochemical properties and soil enzyme activities and to extract total soil DNA for high-throughput sequencing. We found that the diversity of the rhizosphere bacterial community was significantly different between the two intercropping systems. Compared with ZZ1, the ZZ9 intercropping system enriched the nitrogen-fixing bacteria, increasing the available nitrogen content by 18% compared with that with ZZ1. In addition, ZZ9 intercropping with soybean formed a more compact rhizosphere environment than ZZ1, thus providing favorable conditions for sugarcane growth. These results provide guidance for the sugarcane industry, especially for the management of sugarcane and soybean intercropping in Guangxi, China.

## Introduction

Intercropping is simultaneous cultivation of two or more crops in the same field (Esnarriaga et al., 2020). Compared with monocropping, intercropping increases the efficiency of resource utilization and yield, which greatly improves the fertility of agricultural land (Wang Y. et al., 2018). Intercropping commonly involves co-cultivation of soybean and grasses, which increases crop yield through enhanced rhizosphere interaction, improved soil microecology, and increased number of soil microorganisms and enzyme activities (Zhou et al., 2019). Intercropping alters microbial community structure and activity and thereby affects carbon and nitrogen dynamics (Luo et al., 2016; Lian et al., 2018). Lian et al. (2019) reported that sugarcane and soybean intercropping increases microbial diversity in rhizosphere soil and is widely used to stabilize yield and reduce nitrogen leaching. Solanki et al. (2017) demonstrated that sugarcane intercropping with soybean increases the abundance of soil nitrogen-fixing bacteria and the activity of dehydrogenase. Zhang et al. (2021) showed that intercropping systems changed soil microbial numbers and enzyme activities, which in turn regulated genes involved in nitrogen cycle, phosphorus cycle and organic matter turnover. In addition, soybean—related nitrogen fixation improves soil fertility and field ecological conditions, which are beneficial to sugarcane in intercropping systems (Lian et al., 2018), and stimulate nitrogen fixation by the microbiome of legumes (Li et al., 2013).

Microbial communities play an important role in every biogeochemical cycle; are key factors in many ecosystem processes; obtain mineral nutrients such as nitrogen, phosphorus, and sulfur from the soil; and are major contributors to plant growth (Delgado-Baquerizo et al., 2017; Jacoby et al., 2017). By breaking down organic matter, they maintain soil fertility and support sustainable plant growth and productivity (Itelima et al., 2018). Plant growth-promoting rhizobacteria intensify plant growth and affect plant development through a variety of mechanisms, such as formation of iron carriers, phosphate solubilization, biological nitrogen fixation, and production and activation of 1-amino-cyclopropane 1-carboxylic acid deaminase (Xun et al., 2015; Goswami et al., 2016). Biological nitrogen fixation is one of the most important ecological and agricultural benefits of plant–bacterial interaction. The bacterial nitrogenase complex reduces atmospheric N_2_ to ammonia (NH_3_), a form that is easily absorbed by plants (Rilling et al., 2018). Therefore, recruitment of nitrogen-fixing bacteria in symbiotic or non-symbiotic relationships helps host plants obtain nitrogen directly from the atmosphere to meet their nutritional needs (Matos et al., 2021). Some free-living bacterial genera (such as nitrogen-fixing genera) can colonize different plant niches, such as the rhizosphere and endosphere, thereby promoting the nitrogen requirements of non-legumes (Bhattacharyya and Jha, 2012). In addition, phenotypic traits such as root structure affect microbial recruitment and colonization (Saleem et al., 2018). Zhao et al. (2020) demonstrated that sugarcane of different genotypes established different rhizosphere bacterial communities, which was speculated to be related to variation of root exudates.

Sugarcane planting area in Guangxi is an important planting region in China, accountings for 60% of the country’s total sugarcane production and 69% of the country’s total sugar production (Rukai and Zhaonian, 2010). Drought has become the main limiting factor of sugarcane production in this region because of its unique geographical location, uneven distribution of rainfall, and imperfect irrigation facilities (Liu Y. et al., 2020). Therefore, there is an urgent need to breed new drought-resistant and high-yielding sugarcane varieties to improve sugarcane yield. “Zhongzhe” ZZ1 and ZZ9 are two new sugarcane cultivars selected and bred by the State Key Laboratory for Conservation and Utilization of Subtropical Agro-bioresources (Yin et al., 2020). These two cultivars have great advantages in terms of yield and a potential to replace the currently planted sugarcane varieties (Zhang et al., 2019). Previous studies have found differences in water use efficiency between ZZ1 and ZZ9 (Araus et al., 2020), which may also be present during soybean intercropping. The rhizosphere bacterial community structure and nitrogen use in intercropping systems of soybean and these two varieties are largely unknown. In the present study, we evaluated the relationships between the two sugarcane cultivars, ZZ1 and ZZ9, and soybean in intercropping systems through rhizosphere bacterial community diversity analysis, rhizosphere bacterial relative abundance analysis, and correlation network analysis as well as other bioinformatics analysis. The aim of this study was to address the following questions: (1) Does rhizosphere microbial community structure differ between intercropping systems of ZZ1 and ZZ9? (2) Will ZZ1 and ZZ9 enrich nitrogen–fixing bacteria when intercropped with soybean? (3) Which of the two sugarcane–soybean intercropping systems will form a more compact rhizosphere soil environment? This study will provide new ideas for the efficient utilization of sugarcane fields and the green development of the sugarcane planting industry.

## Materials and Methods

### Plants and Field Experimental Design

The study was conducted in the summer of 2018 in Fushun Quli (107°31′–108°06′ E, and 22°17′ –22°57′ N) at a feed breeding farm of the Guangxi University. The average annual temperature in the region was 21.3°C. The annual total radiation was 108.4 Kcal/cm, the annual average sunshine time was 1,693 h, and the frost-free period was 346 days. The annual rainfall in the entire region ranged from 1,050 to 1,300 mm. The soil of the long-term sugarcane field was laterite and had the following characteristics: pH 5.15, organic matter 19.47 g/kg, total nitrogen 0.84 g/kg, total phosphorus 2.98 g/kg, total potassium 7.11 g/kg, alkaline hydrolyzed nitrogen 136 mg/kg, available phosphorus 83 mg/kg, and available potassium 77.1 mg/kg. Two new cultivars, ZZ1 and ZZ9, of the sugarcane cultivar Zhongzhe developed and cultivated in the State Key Laboratory for Conservation and Utilization of Subtropical Agro-bioresources were used in this study. Both cultivars originated from the same parents (Roc 25 × Yunzhe 89-7) (Yin et al., 2020). We used the local soybean cultivar GUIZAO2. This high-yielding cultivar was bred locally in Guangxi for its drought resistance, adaptation to local climate, and shade tolerance. The variety is suitable for intermediate cultivation with sugarcane and is widely used in Guangxi (Huaizhu et al., 2004). Three monoculture (ZZ1, ZZ9, and GUIZAO2) and two intercropping patterns (ZZ1–soybean and ZZ9–soybean) were established, each comprising three plots as replicates (30 m × 42 m). The experimental plots were planted with 12 rows of sugarcane alternating with 12 rows of soybeans at 1.2 m distance (Supplementary Figure 1). Standard agricultural practices for sugarcane planting were followed for field management activities, including irrigation, fertilization, weeding, and pest control.

### Soil Sample Collection and Analysis of Physicochemical Properties

The agronomic characters of sugarcane were measured before harvest, plant height was measured by a steel tape, representative plants were selected from each plot, and the plant height of 20 plants was measured to calculate the average plant height. Plant weight was measured using a weighing balance, according to the empirical formula: single stem weight = plant height × stem diameter × 0.785/1,000. Sugar content was measured using a refractometer (BRIX30, Leica, Bannockburn, IL), and stem weight and sugar content were measured simultaneously with plant height. The roots of each plant were dug out and the loosely attached soil was removed by manual shaking, whereas the rhizosphere soil was collected from the surface of the roots (Wang C. et al., 2018). We collected the rhizosphere soil samples from sugarcane roots in ZZ1 monoculture plots (mono_sug1), ZZ9 monoculture plots (mono_sug9), from ZZ1 and ZZ9 sugarcane roots near the soybean side (inter_sug1 and inter_sug9, respectively), and from soybean roots near the ZZ1 and ZZ9 sugarcane sides (inter_soy1 and inter_soy9, respectively). Six sugarcane and six soybean rhizosphere soil samples were collected from each experimental plot randomly. The roots were shaken vigorously to remove loose soil. The six soil samples from each were then mixed, and plant residues and stones were removed by sieving through a 2 mm sieve. Each soil sample was divided into three parts, which were then used for DNA extraction, determination of environmental factors, and determination of soil enzyme activity. The soil used for DNA extraction and enzyme activity determination was temporarily stored in a refrigerator at 4°C, while the soil used for soil environmental factor determination was air dried at room temperature (25–28°C) and stored in a sealed bag at room temperature. Soil organic carbon (SOC) and available phosphorus (AP) levels were measured as previously described (Soon and Abboud, 1991; Zhaolei et al., 2017). The soil was extracted with 2 M KCl, and the contents of NH_4_-N and NO_3_-N in the filtrate were analyzed using a flow-solution analyzer (Flowsys, Ecotech, Germany). Dissolved nitrogen (DON) and total nitrogen (TN) levels were estimated using a TOC/TN analyzer (Multi N/C 2100 (S); Analytik Jena GmbH, Germany). Levels of soil enzymes were measured using kits for soil urease (S-UE), soil sucrose (S-SC), soil catalase (S-CAT), and soil acid phosphatase (S-ACP) (Wang et al., 2014; Hou et al., 2020). The analysis of each soil sample was repeated three times with 0.5 g per replicate.

### DNA Extraction, Amplicon Generation, and High-Throughput Sequencing

DNA was extracted from the samples using an E.Z.N.A Soil DNA Kit (Omega Bio-Tek, Inc., Norcross, GA, United States). The concentration and purity of the extracted DNA were measured using a NanoDrop One spectrophotometer (Thermo Fisher Scientific, MA, United States). A 50 μL PCR reaction mixture, contained 25 μL of Premix Taq (Takara Biotechnology, Dalian Co., Ltd., Dalian, China), 1 μL each primer (10 mM), and 3 μL template DNA (20 ng/μL), was prepared. The amplification was conducted in a Biorad S1000 Thermal Cycler (Bio-Rad Laboratories, CA, United States) under the following thermal cycling conditions: initial denaturation at 94°C for 5 min, followed by 30 cycles of denaturation at 94°C for 30 s, annealing at 52°C for 30 s, extension at 30°C for 30 s. and final extension at 72°C for 10 min. The primers used were *nifH-F* (AAAGGYGGWATCGGYAARTCCACCAC) and *nifH*-R (TTGTTSGCSGCRTACATSGCCATCAT) (Rösch et al., 2002). The DNA library was constructed using an Illumina TruSeq DNA Sample Preparation Kit (Illumina, San Diego, CA, United States). The Illumina HiSeq2500 platform was used for high-throughput sequencing of the *nifH* genes (Guangdong McGinn Biotechnology Co., Ltd., Guangzhou, China), and the sequences have been deposited in the NCBI database^1^ with accession number PRJNA657992.

### Statistical and Bioinformatics Analysis

Statistical data of soil environmental factors, soil enzyme activities and crop agronomic traits were compared and analyzed using analysis of variance and multiple comparisons using SPSS22.0 (SPSS, Chicago, IL, United States). The raw reads were processed using QIIME2 v.2019.1.0 with DADA2 plug-in to filter low-quality reads, reconstruct the amplicon sequence variants (ASVs), and to generate a feature table for ASV count. In the mass filtering step, the dataset was truncated to a read length of 270–250 bp for forward and reverse reads (all other parameters were set to default values). After mass filtering, the bacterial taxonomy was assigned to the ASV feature table using the naive Bayesian Q2 feature classifier implemented in QIIME2. The data were used for taxonomic classification against the SILVA 132 database for 16S rRNA (Quast et al., 2012) and the *nifH* sequence database (Gaby and Buckley, 2014).

Alpha diversity was analyzed using QIIME2 to assess the complexity of biodiversity in the sample and was represented by R (V3.6.3). Sample diversity was estimated by determining the Chao1 and Shannon indices. At the ASV level, we examined the observed differences in species richness among the three sample types (ZZ1, ZZ9, and GUIZAO2 monocropping) and between the two intercropping patterns (ZZ1–soybean and ZZ9–soybean). Beta diversity analysis was used to assess the differences in sample complexity. For general analysis, the filtered ASV sequence number of each bacterial taxon was normalized using the Bioconductor software package EDGER (version 3.8.5) (Hartman et al., 2018). We used R software and a Local Perl script to create sample removal heat maps based on the UniFrac removal matrix. Principal coordinate analysis (PCoA) was used to show differences in species composition of the microbial community. We examined the effects of sample types and intercropping patterns on community dissimilarity using permutational multivariate analysis of variance. In addition, Pearson’s correlation analysis was used to determine the relationship between alpha diversity and environmental factors and Mantel test was used to examine the relationship between beta diversity and environmental factors. Mantel tests, heatmaps, PCoA, redundancy analysis (RDA), and network analysis were performed using the “vegan” package in R v3.6.3 (Bargaz et al., 2017; Zheng et al., 2019).

The randomForest program in the randomForest package (Grömping, 2009) was used to determine the main prokaryotic indicators of soil microbial community differences in the two intercropping systems by classifying the randomForest model (Jiao et al., 2018). The mean decrease gini values of the variables were used to estimate the relative importance of prokaryotic indicators in determining soil microbial communities, with higher mean decrease gini values indicating that these variables (i.e., the corresponding prokaryote classification) are more important (Grömping, 2009).

### Co-occurrence Network Analysis

The soil and root bacterial communities were further evaluated by determining the Spearman rank correlation coefficients for all bacterial pairs and the topological network attributes. This includes the total number of network nodes (ASVs), the total number of edges (representing the connections between nodes that are significantly positively correlated between ASVs), and the degree of concurrency (the number of direct correlations with nodes). Spearman rank correlation analysis was performed to evaluate the association between all bacterial ASV pairs. We calculated the network properties mentioned above and, in order to investigate the community structure in the rhizosphere networks, we identified the network modules from the substructures of nodes whose group edge densities were higher than those between them (Chun et al., 2020). Microbial taxa that reappeared with other taxa in microbial coexistence networks were considered ecologically significant and were thought to play an important role in the microbiome (Agler et al., 2016; van der Heijden and Hartmann, 2016; Hartman et al., 2018).

## Results

### Comparison of Nitrogen-Fixing Microbial Diversity Between Two Intercropping Patterns

**FIGURE 1:**
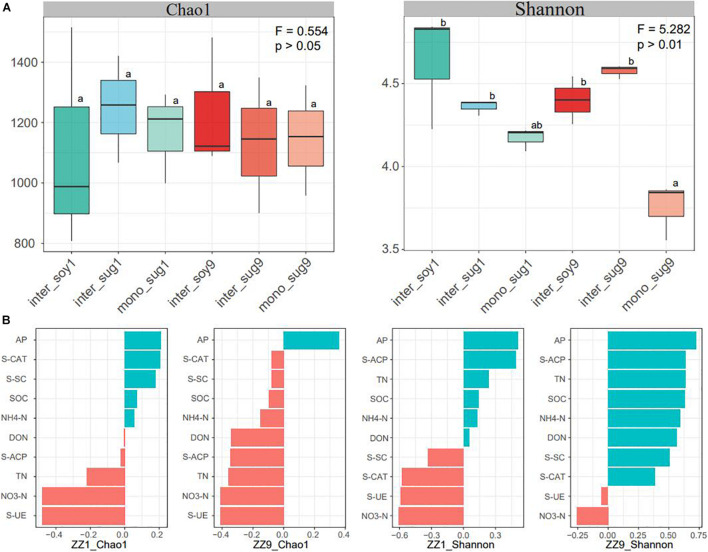
**(A)** Bacterial alpha-diversity measurements of represented by Chao 1 and Shannon indexes in each pattern. F = Fisher’s F-ratio. *p* = *p*-value. Different letters next to the bars represent significant differences between the measured indices. **(B)** Pearson correlation analysis of alpha diversity and environmental factors showed positive correlation in blue and negative correlation in red.

**FIGURE 2:**
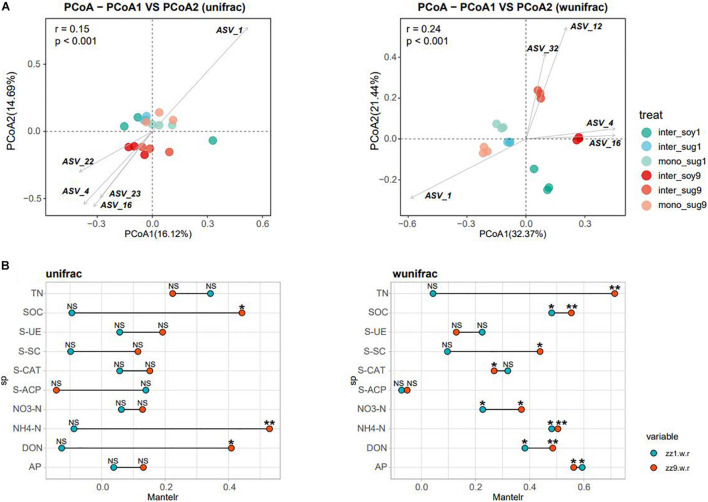
**(A)** Principal coordinate analyses (PCoAs) using UniFrac distance matrix, to analyze the differences in the diversity of bacterial beta under different patterns, figure in left side is unweighted, figure in right side is weighted. **(B)** Mantel test was used to o compare the correlation difference of soil nutrients between ZZ1 and ZZ9, figure in left side is unweighted, and figure in right side is weighted. *P* > 0.05 (NS), *P* < 0.05(*), *P* < 0.01(**).

Alpha diversity results showed no significant difference in the Chao1 index between the two intercropping patterns. The Shannon index of inter_soy and inter_sug of the two intercropping patterns was higher than mono_sug, and the difference was more significant in the ZZ9–soybean intercropping pattern (Figure 1A). Pearson’s correlation analysis of the alpha diversity and environmental factors revealed that the Chao1 index of ZZ9 was negatively correlated with more environmental factors than that of ZZ1 (Figure 1B). The correlation degree of Chao1 index with S-CAT, S-SC, SOC, NH4-N, DON, and S-ACP was significantly different between the two intercropping systems. In terms of Shannon index, ZZ9 was positively correlated with significantly greater number of environmental factors than ZZ1, and it had a higher positive correction in AP, TN, SOC, NH4-N, S-ACP, and DON than ZZ1 (Figure 1B). Beta diversity analysis was based on weighted and unweighted UniFrac, reflecting the differences in the bacterial community structure of each treatment (Figure 2A). Under the unweighted condition, the distance between the components (inter_soy, inter_sug, and mono_sug) in the two intercropping patterns was not large, and the community differences between the inter_soy, inter_sug, and mono_sug in ZZ1–soybean intercropping system was not obvious. In the ZZ9–soybean intercropping system, the community structure of inter_soy and inter_sug was obviously different from that in mono_sug and significantly different from those in the ZZ1 interculture system (Figure 2A). These differences were mainly influenced by ASV1 (*Burkholderia*), ASV4 (*Desulfopila*), ASV16 (*Aquabacterium*), ASV22 (Clostridiales unclassified), and ASV23 (*Aquabacterium*). Under weighted UniFrac, considering the effect of population abundance, the differences between composition of the components in the two intercropping patterns were obvious (Figure 2A). In the ZZ1–soybean intercropping system, the difference between inter_sug and mono_sug components was small, and both were significantly different from that of inter_soy. In the ZZ9–soybean intercropping system, the composition of inter_soy, inter_sug and mono_sug was significantly different. The difference between the two intercropping patterns was mainly influenced by ASV1 (*Burkholderia*), ASV4 (*Desulfopila*), ASV12 (*Terrimicrobium*), ASV16 (*Aquabacterium*), and ASV32 (*Methylocystis*). The Mantel test of beta diversity and environmental factors showed that, without weighting, the diversity indices of ZZ1 and ZZ9 were significantly different in correlation with SOC, NH_4_-N, and DON, with ZZ9 being highly significant in these three environmental factors. After weighting, the two intercropping patterns were mainly differed in correlation with TN and S-SC, and ZZ9 exhibited a very significant difference in TN (Figure 2B).

### Nitrogen-Fixing Bacterial Community Changes and Their Relationship With Environmental Factors

**FIGURE 3:**
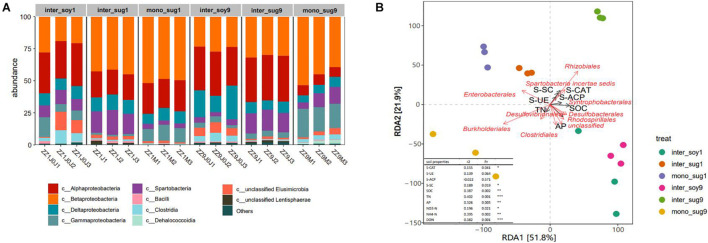
**(A)** Relative abundance of different phyla in each pattern. **(B)** Distance-based redundancy analysis of different patterns (dot) and environmental factors (arrows) indicates the dominant communities and influential environmental factors, Pr ≤ 0.001(***), Pr ≤ 0.01(**), * Pr ≤ 0.05(*).

Changes in bacterial community composition in the two intercropping patterns at the phylum level were analyzed. In the ZZ1–soybean intercropping system, the relative abundance of Spartobacteria in inter_sug1 was significantly higher than that in mono_sug1, whereas the relative abundance of Gammaproteobacteria decreased. In the ZZ9–soybean intercropping system, the relative abundance of Betaproteobacteria and Gammaproteobacteria were significantly higher and that of Alphaproteobacteria was significantly lower in inter_sug9 than in mono_sug9 (Figure 3A). Similarly, the relative abundance of Alphaproteobacteria was higher and that of Betaproteobacteria was lower in inter_sug9 than in inter_sug1. We used RDA to explore the correlation between bacterial composition and environmental factors. The first two axes of RDA accounted for 51.8 and 21.9% of the total data variation (Figure 3B). In the ZZ1 intercropping system, the community structures of mono_sug1 and inter_sug1 were more similar to those of inter_soy1; the main influencing factors were AP and SOC, and the difference was attributed to Enterobacterales, Desulfobacterales, Clostridiales, and Rhodospirillale. The community structure of each component was significantly different in ZZ9 intercropping system. The differences between inter_soy9 and inter_sug9 were mainly influenced by AP, Clostridiales, Rhodospirillales, and Desulfobacterales. The differences between inter_sg9 and mono_sug9 were mainly derived from S-SC and S-CAT, and were strongly correlated with Rhizobiales and Burkholderiales. The differences between 1inter_sug and 9inter_sug were mainly influenced by S-SC and S-CAT, and were strongly related to Rhizobiales and Burkholderiales.

**FIGURE 4:**
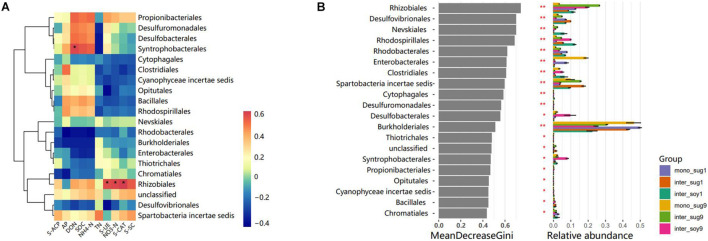
**(A)** Pearson correlation heat maps of environmental factors and relative abundances of the 20 most abundant bacterial genera in rhizosphere soil samples. **(B)** Top 10 bacterial genera and their mean decrease in Gini, which is a measure of characteristic correlation. The higher the index of mean decrease Gini, the more important the bacterial genera are.

The correlation heat map of different phyla with environmental factors showed a significantly positive correlation of Rhizobiales with S-UE, NO3-N and S-CAT, in addition, there was a significantly positive correlation in Syntrophobacterales and DON (Figure 4A). Based on the constructed random forest model, the mean decrease in Gini identified Rhizobiales, Desulfovibrionales, and Nevskiales as the three most important orders in microbial communities (Figure 4B). In addition, Rhizobiales and Burkholderiales had high relative abundances in each component.

### Co-occurrence Network Analysis

**FIGURE 5:**
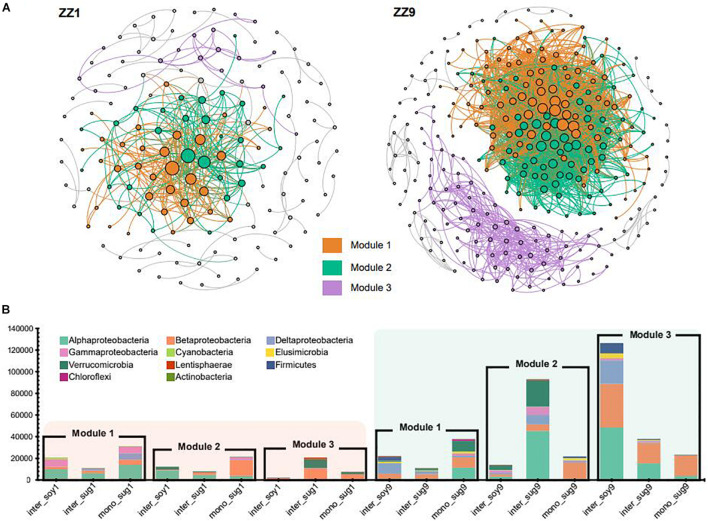
**(A)** Significant correlation of co-occurrence network visualization (ρ > 0.7, *P* < 0.001; In ZZ1 and ZZ9 communities, bacterial ASVs were represented by a gray line. Dots represent an ASV, and ASVs are colored according to their association with different cropping systems (gray ASVs are insensitive to variety differences). The size of the dots reflects the association between ASVs and other ASVs, and the more associated ASVs the larger the dots are **(B)** cumulative relative abundance in different cropping systems, and different colors represent different phyla.

The co-occurrence network analysis of ASVs in the two intercropping patterns identified the same modules, namely, Module1 (M1), Module2 (M2), and Module3 (M3). However, the modules in the ZZ9–soybean intercropping system were more closely related and exhibited more ASV associations than those in the ZZ1 intercropping system (Figure 5A). The correlation heat map between each module and environmental factors in the two intercropping patterns showed that in the ZZ1–soybean intercropping system, M1 was significantly negatively correlated with AP, and significantly positively correlated with S-CAT; M2 was significantly negatively correlated with AP, and positively correlated with S-CAT; and M3 was significantly positively correlated with S-SC (Supplementary Figure 3). In the ZZ9–soybean system, M3 was significantly positively correlated with TN; M1 and M3 were significantly negatively correlated with NH_4_-N, SOC, and DON; and M2 was significantly negatively correlated with NH_4_-N. Supplementary Figure 2 showed the values of the intra and inter-module connectivity of the two intercropping patterns. Almost all connectors and module hubs, considered keystones, existed in the network of ZZ9, while their number in ZZ1 was very small. Analysis of the number of ASVs of each module in the co-occurrence network showed that, in the ZZ1–soybean intercropping system, the numbers of ASVs contained in M1, M2, and M3 modules were not high, with Alphaproteobacteria being dominant in M1 (Figure 5B). M2 mainly contained ASVs in mono_sug1 and was dominated by Betaproteobacteria. In M3, ASVs were mainly in inter_sug1, and Betaproteobacteria and Verrucomicrobia were dominant. In the ZZ9–soybean intercropping system, three modules contained a large number of ASVs, and M2 mainly corresponded to inter_sug9, which contained a large number of Alphaproteobacteria and Verrucomicrobia. The ASVs in M3 were mostly from inter_soy9 and contained a large number of Alphaproteobacteria, Betaproteobacteria, and Deltaproteobacteria.

### Comparison of Nutrients, Enzymes, and Agronomic Traits in the Field

**FIGURE 6:**
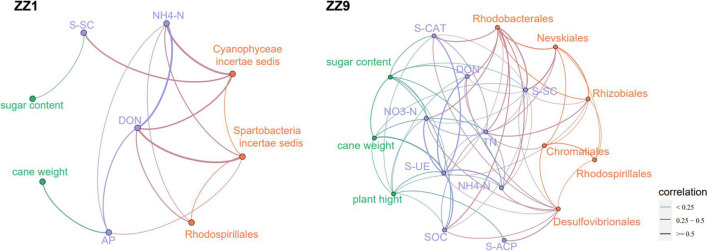
The correlation network analysis of agronomic traits, environmental factors and bacterial genera between the two sugarcane cultivars, the thickness of the line reflects the correlation.

Compared with mono_sug1, inter_sug1 increased cane weight, plant height, and sugar content, but the differences were not significant. Compared with mono_sug9, plant height, cane weight, and sugar content of inter_sug9 were significantly increased. The promoting effect of ZZ9–soybean intercropping on the three agronomic traits was more significant than that of intercropping with ZZ1 (Table 1). The results of the analyses of soil environmental factors and enzyme activities showed that inter_sug9 had higher enzyme activities than inter_sug1, and S-CAT, S-UE, and S-SC and the contents of TN and NO_3_-N were also higher in inter_sug9 than in inter_sug1 (Table 2). ZZ9 had a stronger correlation with sugarcane agronomic traits, environmental factors, and keystones than ZZ1 (Figure 6). In ZZ1, sugarcane cane weight was correlated only with AP, sugar content was correlated only with S-SC, DON was highly correlated with NH_4_-N and Spartobacteria *incertae sedis*, and NH_4_-N was highly correlated with Cyanophyceae *incertae sedis*. In ZZ9, the correlation between each index was larger than that in ZZ1. S-UE was highly correlated with cane weight, NO_3_-N, SOC, NH_4_-N, DON, and S-CAT, whereas S-SC was strongly correlated with Rhodobacterales and Nevskiales.

**TABLE 1:** Agronomic characters of sugarcane.

Sample name	Plant height (mm)	Cane weight (kg)	Sugar content %
inter_sug1	267.50 ± 3.04b	1.91 ± 0.09b	21.78 ± 0.81b
inter_sug9	322.83 ± 1.76a	2.31 ± 0.10a	24.53 ± 0.60a
mono_sug1	263.17 ± 13.71bc	1.17 ± 0.48b	20.87 ± 1.02b
mono_sug9	244.17 ± 7.52c	1.38 ± 0.19c	22.48 ± 0.58a
Pr(> F)	<0.01	0.243	0.239

*Different letters indicate significant differences (ANOVA, P < 0.05).*

**TABLE 2:** Soil enzyme activity and environmental factors.

Treatment	S-CAT	S-UE	S-ACP	S-SC	SOC	TN	AP	NO3-N	NH4-N	DON
inter_soy1	10.96 ± 0.60a	294.34 ± 34.90ab	56.15 ± 5.22a	54.38 ± 0.31a	14.14 ± 0.87ab	1.22 ± 0.07ab	1.80 ± 0.32bc	8.96 ± 0.16ab	2.24 ± 0.11ab	26.83 ± 1.42ab
inter_sug1	11.31 ± 0.66a	252.87 ± 28.79a	56.07 ± 5.46a	54.84 ± 0.24a	18.22 ± 1.09cd	1.18 ± 0.06a	1.89 ± 0.04bc	8.79 ± 0.12a	2.71 ± 0.13cd	31.37 ± 1.51bc
mono_sug1	12.45 ± 0.41a	363.53 ± 46.29b	53.82 ± 2.15a	54.58 ± 0.19a	11.19 ± 0.02a	1.12 ± 0.07a	1.02 ± 0.34a	9.29 ± 0.22bc	1.89 ± 0.00a	23.91 ± 0.46a
inter_soy9	11.47 ± 2.20a	382.01 ± 37.77b	57.00 ± 4.15a	54.55 ± 0.83a	21.22 ± 1.99d	1.92 ± 0.03d	2.00 ± 0.96c	9.38 ± 0.15d	3.07 ± 0.24c	36.35 ± 2.85bc
inter_sug9	22.49 ± 2.08b	487.65 ± 42.25c	62.41 ± 3.88a	93.01 ± 1.61c	17.59 ± 1.75bc	1.55 ± 0.00c	1.56 ± 0.01b	11.65 ± 0.15d	2.78 ± 0.21d	31.90 ± 2.61bc
mono_sug9	11.54 ± 1.13a	363.90 ± 27.08b	57.61 ± 4.06a	62.47 ± 5.03b	14.48 ± 0.96ab	1.37 ± 0.10b	1.82 ± 0.03bc	9.66 ± 0.23c	2.32 ± 0.12bc	27.75 ± 1.25ab
Pr(> F)	<0.01	<0.01	0.558	<0.01	<0.01	<0.01	<0.01	<0.01	<0.01	<0.01

*Different letters indicate significant differences (ANOVA, P < 0.05).*

## Discussion

### The Two Intercropping Patterns Formed Different Rhizosphere Bacterial Community Structures

The Shannon index of inter_soy and inter_sug of the two intercropping systems was significantly higher than that of mono_sug (Figure 1A), suggesting that the intercropping of sugarcane and soybean improved the diversity of rhizosphere bacteria (Li and Wu, 2018). It is worth noting that the Shannon index of the ZZ9–soybean intercropping pattern was significantly higher than that of the ZZ9 monocropping. One possible explanation for this significant increase in diversity may be related to the greater amount and type of root exudates in the intercropping system than in the monocropping system (Zhou et al., 2011). The release of these exudates into the rhizosphere soil increases soil nutrients and provides a substrate conducive for bacterial growth (Ren et al., 2016; Qiao et al., 2017). ZZ9 was significantly positively correlated with all environmental factors except S-UE and NO3-N (Pearson’s correlation coefficients above 0.5) (Figure 1B), indicating a close correlation between the rhizosphere bacterial community of ZZ9 and the soil environment. The complex interaction between the bacterial community and the environment can establish a stable metabolic environment for the bacterial community, which is conducive to nitrogen fixation (Liu H. et al., 2020).

The beta diversity analysis showed that the bacterial communities of each component under the two intercropping patterns were significantly different, especially among inter_soy9, inter_sug9, and other components (Figure 2A). The unique rhizosphere bacterial community structure produced by ZZ9 intercropping with soybean reflects the strong influence of ZZ9 on the rhizosphere. The effect of plant roots on rhizosphere bacterial composition has been widely recognized in many plant species (Lundberg et al., 2012; Peiffer et al., 2013; Edwards et al., 2015), as well as in sugarcane (Liu et al., 2019). It is worth noting that the difference in the components of the two intercropping modes is mainly driven by Rhizobiales, Burkholderiales, and Desulfobacterales (Figure 2A and Supplementary Table 1), the orders harboring bacteria with recorded or potential N cycle function in the soil (Van Der Heijden et al., 2008; Tang et al., 2018; Nie et al., 2021). The RDA analysis also confirmed the impact of these bacteria on each component in the two intercropping patterns (Figure 3B). This rhizosphere effect may be influenced by the specific root exudates of ZZ9 (Liu Y. et al., 2020). The rhizosphere soil samples mono_sug1 and mono_sug9 showed significant differences in SOC content (Table 2). The activity of various soil enzymes was higher in the ZZ9–soybean intercropping. Soil enzyme activities reflect the rate of soil nutrient cycling and utilization and may be an indicator of soil biodiversity, productivity, and potential microbial activities (Williams and Jochem, 2006). Higher soil enzyme activity may indicate that more metabolic substrates being released into the soil (Yi et al., 2018), thus providing richer soil nutrients environments for bacterial community establishment. Therefore, we conclude that the difference in rhizosphere bacterial community structure between the two intercropping modes is caused by the difference in soil nutrient environment created by root exudates released by the intercropped plants (el Zahar Haichar et al., 2008; Berg and Smalla, 2009).

### Zhongzhe9–Soybean Intercropping Pattern Enriches the Nitrogen-Fixing Soil Bacterial Community

The relative abundance of Alphaproteobacteria in ZZ9–soybean intercropping was higher than that of ZZ1 intercropping mode (Figure 3A). Alphaproteobacteria includes a large number of symbiotic nitrogen-fixing bacteria in soil (Wang et al., 2019). FixK and FnrN in Alphaproteobacteria regulate the expression of oxidase, hydrogen uptake, nitrogen metabolism, heme biosynthesis, and the levels of transcription factors involved in nitrogen fixation (Tsoy et al., 2016). Interestingly, the relative abundance of Deltaproteobacteria was higher in inter_soy9 than in inter_soy1 (Figure 3A). Masuda et al. (2017) showed that diazotrophs harboring nitrogenase (Nif) can drive nitrogen fixation and that the taxonomic composition of Nif transcripts was dominated by Deltaproteobacteria. This suggests that Deltaproteobacteria plays a key role in nitrogen fixation. The importance of each order in the present study was estimated by calculating the mean decrease of Gini index for each order using the random forest algorithm. The importance of Rhizobiales was the highest, and the relative abundance of each component corresponding to the ZZ9 intercropping pattern was higher than that of ZZ1 intercropping pattern (Figure 4B). The growth-promoting role of Rhizobiales has been extensively studied (Garrido-Oter et al., 2018). By providing precursors of various nutrients, plant hormones, and essential plant metabolites, Rhizobiales perform beneficial functions for their hosts, and the most important function is nitrogen fixation (Ivanova et al., 2000; Delmotte et al., 2009; Verginer et al., 2010). We found a significant positive correlation between the activity of Rhizobiales and S-UE and NO3-N (Figure 4B). Urease can hydrolyze urea in the soil and convert it into ammonia, which can be used by plants (Wang et al., 2014), and its activity plays an important role in the nitrogen cycle (Blagodatskaya and Kuzyakov, 2008). Recent studies have shown that nitrogen fixation by rhizobia is not limited to legumes (Pold et al., 2018; Noh et al., 2019), but is found in other non-leguminous higher plants, including sugarcane (Zhao et al., 2020). In addition, the relative abundance of Burkholderiales was high (Figure 4B), and Burkholderiales have been shown to play an important role in nitrogen input to newly colonized soils (Ortiz et al., 2020). In the cluster modules of the co-occurrence network of the two intercropping patterns, the number of ASVs was greater and Alphaproteobacteria, Betaproteobacteria, and Verrucomicrobia were more abundant in the ZZ9 than in the ZZ1 intercropping system (Figure 5B). These bacteria have a positive effect on nitrogen fixation and contributed to the significant increase in TN, NO_3_-N, NH_4_-N, and DON contents in the sugarcane rhizosphere of the ZZ9 intercropping system when compared with ZZ1 system (Table 2 and Supplementary Figure 3; Kämpfer et al., 2008; Khadem et al., 2010; Stein, 2018).

### Intercropping of Zhongzhe9 With Soybean Creates a More Compact Rhizosphere Environment

The results of the alpha diversity analysis confirmed that the rhizosphere bacterial diversity of the ZZ9 intercropping system was strongly positively correlated with multiple environmental factors, including TN, NH4-N, and DON (Figure 1B), and the bacterial relative abundance was significantly different between the two intercropping patterns (Figure 3A). These results indicate that ZZ9 intercropping with soybean established specific bacterial communities in the rhizosphere soil, where these bacteria played an important role in changing the rhizosphere soil microenvironment. This change is closely related to the production of crop root exudates (Semchenko et al., 2014). Bacterial colonization in the rhizosphere is related to root exudates (Lugtenberg and Kamilova, 2009), which act as signals mediating root–microbial interactions (Ling et al., 2011). Bacterial communities are affected by root exudates, which alter soil nutrition, help enrich microorganisms, and promote biofilm formation or act as chemical stimulants of bacterial growth (Zhang et al., 2014; Carvalhais et al., 2015; Haldar and Sengupta, 2015). Network analysis has been used to study microbial symbiosis in many complex environments (Barberán et al., 2012). Co-occurrence patterns are important for understanding microbial community structure, and they provide new insights into the underlying networks of interactions, revealing niche spaces shared by community members (Wu et al., 2019). In this study, the ZZ9 intercropping mode established more close connections among ASVs in the network than ZZ1 (Figure 5A). Modularity is characteristic of large complex systems (Barabasi and Oltvai, 2004; Newman, 2006). In a biological network, highly interconnected species are grouped into a module in which interactions between species are more frequent and intensive than that in other members of the community (Barabasi and Oltvai, 2004). Moreover, many environmental factors in ZZ9 intercropping mode are closely related to Rhizobiales, Rhodobacterales and Desulfovibrionales (Figure 6). Previous studies have suggested that Rhizobiales are the main promoters of plant nitrogen fixation, root nodules formations and crop growth (Turan et al., 2019), while Rhodobacterales and Desulfovibrionales have been shown to contribute to nitrogen fixation in rhizosphere soil (Blumenberg et al., 2012; Tsoy et al., 2016). We believe that the intercropping of ZZ9 and soybean establishes a more compact bacterial environment, in which the relationships between bacteria and soil environment are more complex, and the ecosystem is more stable than that in ZZ1 intercropping pattern (Yang et al., 2018). Weidner et al. (2015) suggested that high soil microbial diversity is conducive to positive plant–soil feedback and nitrogen supply in the soil. Differences in biomass and root morphology among different varieties of the same species may lead to differences in microbial diversity and abundance (Hui et al., 2017; Chen et al., 2019). ZZ9 showed better agronomic traits than ZZ1 (Table 1), which indicated that the sugarcane strains had different degrees of interaction with soybean in intercropping. Plant–plant interactions shape the ecosystem structure by changing the community composition (Kardol et al., 2010). The intercropping of ZZ9 and soybean improved the nutrient environment of rhizosphere soil, formed a different bacterial community from that in ZZ1–soybean intercropping system, and further enriched Rhizobiales, Rhodobacterales, and Desulfovibrionales, the orders with a positive effect on nitrogen fixation ultimately improving the absorption and utilization of nitrogen by plants (Liu Y. et al., 2020).

## Conclusion

This study described the differences in bacterial community diversity and composition between sugarcane cultivars ZZ1 and ZZ9 and soybean, as well as their effects on soil environmental factors and agronomic traits of the sugarcane crops. Using the 16S rRNA sequencing data, we found that the bacterial community structure of the ZZ9–soybean intercropping system was significantly different and highly diverse compared with that of the ZZ1 intercropping system. In addition, in the sugarcane-soybean intercropping system, ZZ9 accumulated more nitrogen-fixing bacteria, and had a closer relationship with the soil environment than ZZ1. Therefore, the interaction between ZZ9 and legumes can potentially promote efficient agricultural production, which is of great significance to the sugarcane industry in Guangxi, China. In addition, our findings will be helpful to the design of intercropping systems and the selection of the best varieties, to enrich the bacterial community structure and create an environment conducive to crop growth, thus maximizing crop yield.

## Data Availability Statement

The datasets presented in this study can be found in online repositories. The names of the repository/repositories and accession number (s) can be found below: https://www.ncbi.nlm.nih.gov/, PRJNA657992.

## Author Contributions

YL: writing-original draft preparation and investigation. WM: methodology, investigation, and formal analysis. HH: software and validation. YC: visualization, data curation, and writing—review and editing. ZW: writing—review and editing, conceptualization, and software. All authors contributed to the article and approved the submitted version.

## Conflict of Interest

The authors declare that the research was conducted in the absence of any commercial or financial relationships that could be construed as a potential conflict of interest.

## Publisher’s Note

All claims expressed in this article are solely those of the authors and do not necessarily represent those of their affiliated organizations, or those of the publisher, the editors and the reviewers. Any product that may be evaluated in this article, or claim that may be made by its manufacturer, is not guaranteed or endorsed by the publisher.

## Footnotes

1
http://www.ncbi.nlm.nih.gov

## Supplementary Material

Supplementary Figure 1 Diagram of planting pattern.

Supplementary Figure 2 The inter module and intra module connectivity values of the two intercropping modes. Dots represent connectors, triangles represent module hubs, squares represent peripheral nodes, and colors represent different phyla, and size represent the relative abundance.

Supplementary Figure 3 Pearson correlation analysis between modules in co-occurrence network analysis and environmental factors.
