# Supplementary material for: Effects of Sugarcane and Soybean Intercropping on the Nitrogen-Fixing Bacterial Community in the Rhizosphere
Source: Front Microbiol. 2021 Sep 30;12:713349. doi: 10.3389/fmicb.2021.713349 (PMC8515045; doi:10.3389/fmicb.2021.713349)
Supplement: Supplementary Figure 1 — Diagram of planting pattern. [file Image_1.pdf]

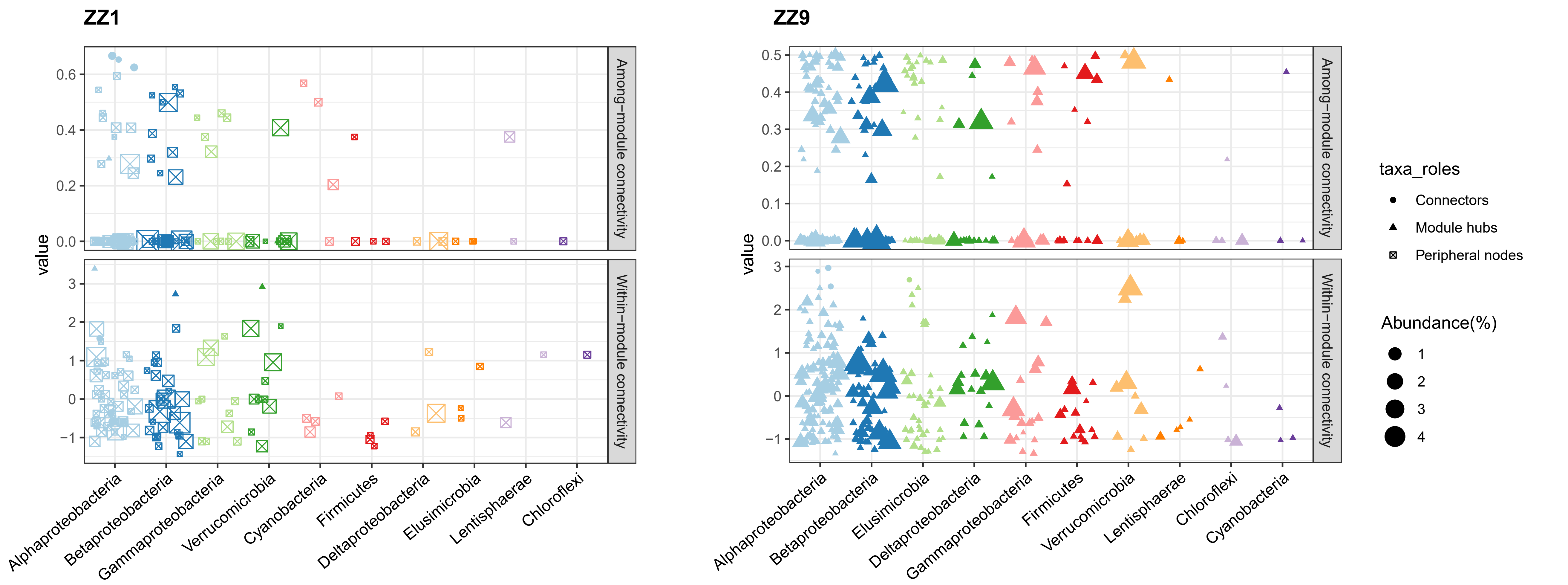

ZZ1
ZZ9
Among−module connectivity
Within−module connectivity
value
0.0
0.2
0.4
0.6
−1
0
1
2
3
0.1
0.3
0.5
Alphaproteobacteria
Betaproteobacteria
Gammaproteobacteria
Verrucomicrobia
Cyanobacteria
Firmicutes
Deltaproteobacteria
Elusimicrobia
Lentisphaerae
Chloroflexi
taxa_roles
Connectors
Module hubs
Peripheral nodes
Abundance(%)
1
2
3
4
