# Supplementary figures and images for: Effects of Sugarcane and Soybean Intercropping on the Nitrogen-Fixing Bacterial Community in the Rhizosphere
Source: Front Microbiol. 2021 Sep 30;12:713349. doi: 10.3389/fmicb.2021.713349 (PMC8515045; doi:10.3389/fmicb.2021.713349)

**ZZ1**

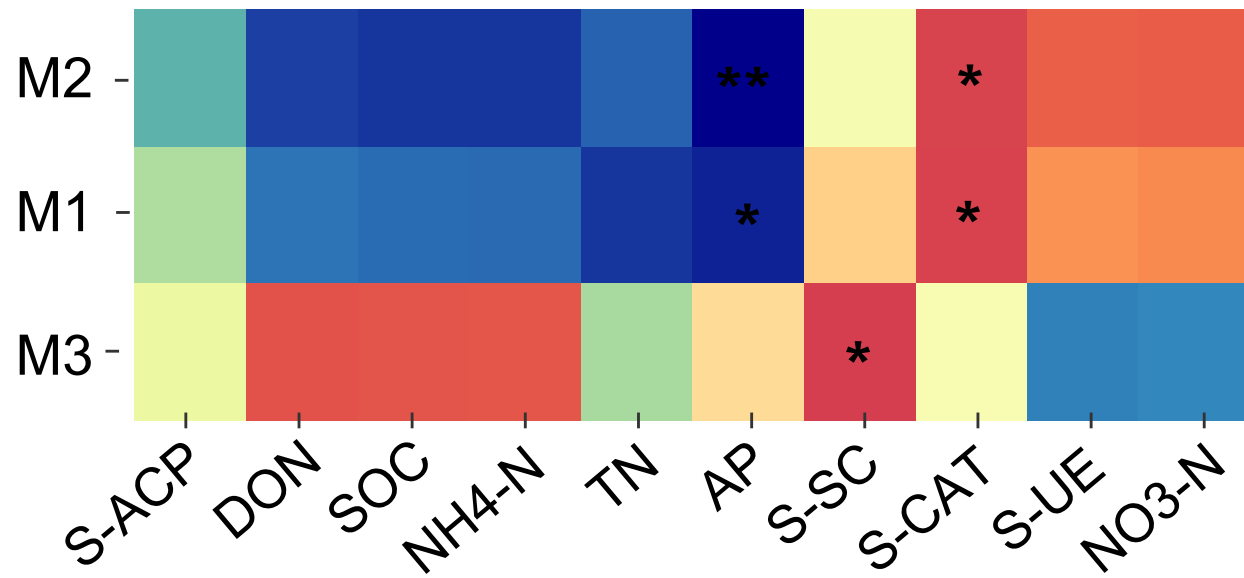

**ZZ9**

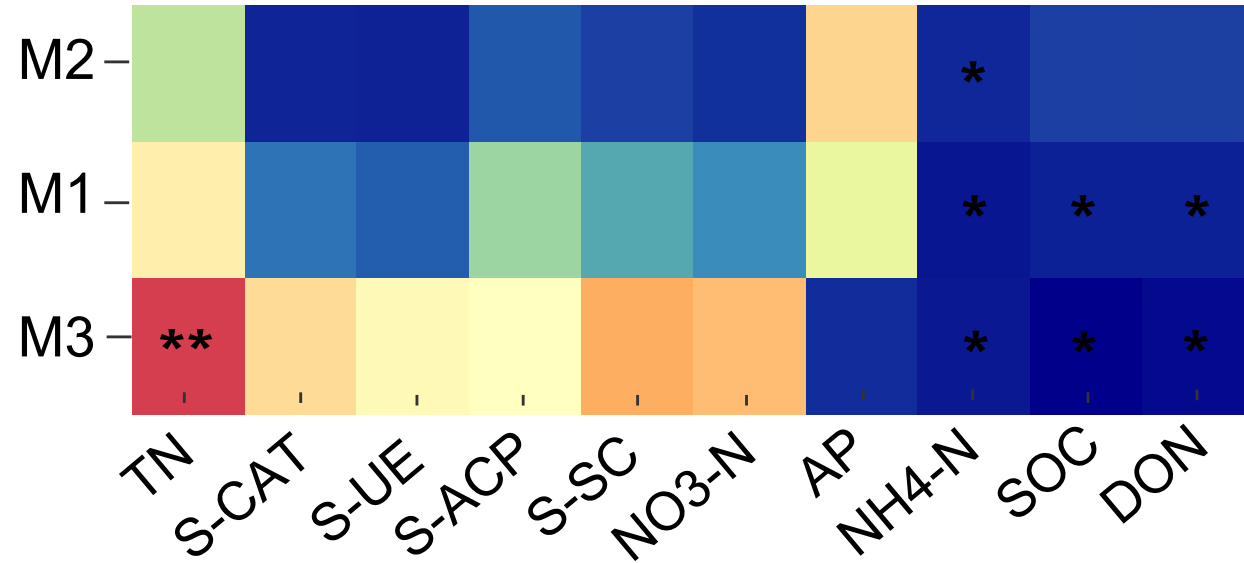

pearson

0.5

0.0

-0.5

Supplement: Supplementary Figure 2 — The inter module and intra module connectivity values of the two intercropping modes. Dots represent connectors, triangles represent module hubs, squares represent peripheral nodes, and colors represent different phyla, and size represent the relative abundance. [file Image_2.pdf]

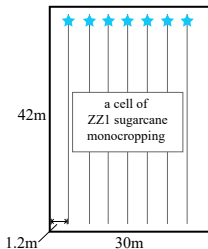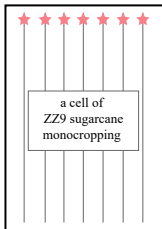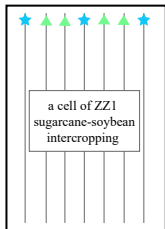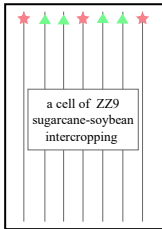

### Legends

- ★ — ZZ1 sugarcane line
- ★ — ZZ9 sugarcane line
- ▲ — soybean line

Supplement: Supplementary Figure 3 — Pearson correlation analysis between modules in co-occurrence network analysis and environmental factors. [file Image_3.pdf]
