# Supplementary material for: Effects of Sugarcane and Soybean Intercropping on the Nitrogen-Fixing Bacterial Community in the Rhizosphere
Source: Front Microbiol. 2021 Sep 30;12:713349. doi: 10.3389/fmicb.2021.713349 (PMC8515045; doi:10.3389/fmicb.2021.713349)
Supplement: Supplementary file 4 [file Table_1.docx]

| **Table S1** | | | | | | |
| --- | --- | --- | --- | --- | --- | --- |
| **ASV** | **Kingdom** | **Phylum** | **Class** | **Order** | **Family** | **Genus** |
| ASV_22 | Bacteria | Firmicutes | Clostridia | Clostridiales | Clostridiales Family XI Incertae Sedis | unclassified |
| ASV_4 | Bacteria | Deltaproteobacteria | Deltaproteobacteria | Desulfobacterales | Desulfobulbaceae | Desulfopila |
| ASV_23 | Bacteria | Betaproteobacteria | Betaproteobacteria | Burkholderiales | Burkholderiales incertae sedis | Aquabacterium |
| ASV_16 | Bacteria | Betaproteobacteria | Betaproteobacteria | Burkholderiales | Burkholderiales incertae sedis | Aquabacterium |
| ASV_1 | Bacteria | Betaproteobacteria | Betaproteobacteria | Burkholderiales | Burkholderiaceae | Burkholderia |
| ASV_12 | Bacteria | Verrucomicrobia | Spartobacteria | Spartobacteria incertae sedis | Spartobacteria incertae sedis | Terrimicrobium |
| ASV_32 | Bacteria | Alphaproteobacteria | Alphaproteobacteria | Rhizobiales | Methylocystaceae | Methylocystis |
